# Ononin alleviates DSS‐induced colitis through inhibiting NLRP3 inflammasome via triggering mitophagy

**DOI:** 10.1002/iid3.776

**Published:** 2023-02-09

**Authors:** Ting Yu, Xuejia Lu, Yan Liang, Lin Yang, Yuehan Yin, Hong Chen

**Affiliations:** ^1^ Department of Gastroenterology, Zhongda Hospital, School of Medicine Southeast University Nanjing Jiangsu People's Republic of China; ^2^ China HuaYou Group Corporation Beijing People's Republic of China

**Keywords:** colitis, mitochondrial damage, mitophagy, NLRP3 inflammasome, Ononin

## Abstract

Background: Ononin, a flavonoid isolated from *Astragalus membranaceus* root, is the active ingredient of *A. membranaceus* and has potential anti‐inflammatory properties, but its effect on colitis is unclear. Aims: This study aimed to explore the anticolitis effect of Ononin by establishing a colitis model in mice induced by dextran sulfate sodium (DSS). Methods: Male C57BL/6 mice were provided DSS, then treated with Ononin (10, 20, 40 mg/kg) or 5‐ASA (40 mg/kg). The colitis symptoms were observed, the disease activity index (DAI) score were recorded daily, and colonic inflammation was evaluted by histopathological scoring. The expression of cytokines, inflammatory mediators, and mitophagy/NLRP3 inflammasome‐related proteins were measured. Results: Ononin significantly alleviated weight loss and colon shortening in mice with colitis (*p* < .01). Moreover, Ononin decreased the production of inflammatory cytokines and mediators associated with colitis (*p* < .05). In addition, Ononin inhibited macrophages infiltration and reduced caspase‐1 activation in colitis mice. Caspase‐1 activation is closely related to the NLRP3 inflammasome. Therefore, we investigated the effect of Ononin on NLRP3 inflammasome in vitro. The relevant results confirmed that Ononin inhibited NLRP3 inflammasome activation and inhibited mitochondrial damage (*p* < .05). Further studies revealed that Ononin inhibited mitochondrial damage through triggering mitophagy (*p* < .05). Conclusion: Ononin alleviates DSS‐induced colitis by activating mitophagy to inhibit NLRP3 inflammasome.

## Graphical abstract

Ononin obviously relieved dextran sulfate sodium‐induced colitis symptoms. Ononin inhibited NLRP3 inflammasome activation. Ononin suppressed NLRP3 inflammasome activation by limiting damaged mitochondria clearance, mediated by PRKN‐dependent mitophagy.

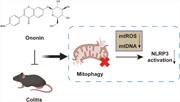

## 1 INTRODUCTION

Inflammatory bowel disease (IBD) is one kind of chronic recurrent inflammation, including ulcerative colitis and Crohn's disease.^1^ IBD patients develop mucosal ulcers, epithelial barrier destruction, rectal bleeding, and diarrhea,^2^, ^3^, ^4^ even enhancing the risk of colon cancer. Current studies cannot elucidate the exact pathogenesis of IBD. However, many studies have clarified that the production of proinflammatory cytokines in the mucosal immune system plays a crucial role in IBD.^5^ More importantly, a variety of cytokines are secreted by a large number of macrophages of IBD patients, such as interleukin IL‐1β, IL‐6, IL‐18, and TNF‐α. All of these factors enhance the intensity of the inflammatory response and increase the severity of colitis.^6^

Inflammasome is an important component of innate immune defense. In response to cellular infection or stress, protein complexes containing NOD‐like receptors (NLRs) are activated to form inflammasomes.^7^ Among them, NLRP3 inflammasome has been studied in the most detail, which is related to the pathogenesis of inflammatory diseases, including IBD.^8^ Activation of NLRP3 inflammasome promotes the maturation of IL‐1β and IL‐18, which is significantly enhanced during the progression of IBD.^9^ In addition, genome‐wide association studies (GWAS) analyses report the NLRP3 gene's association with IBD.^10^ Therefore, it is essential for the limitation of intestinal inflammation and colon cancer development in IBD progression to target the pathogenic role of the NLRP3 inflammasome.

NLRP3 inflammasome agonists are diverse, such as adenosine triphosphate (ATP), bacterial toxins, and microcrystalline substances.^11^ More and more studies have confirmed that different NLRP3 agonists can cause mitochondrial damage, resulting in the release of fragmented mitochondrial DNA (mtDNA) and mitochondrial reactive oxygen species (mtROS).^12^ Therefore, alleviating mitochondrial damage prevents activation of NLRP3 inflammasome. In the process of regulating mitochondrial homeostasis, mitophagy is initiated to degrade damaged mitochondria.^13^ Damaged mitochondria are ubiquitinated by the E3 ubiquitin ligase Parkin, triggering translocation of ubiquitin‐binding autophagy receptors to mitochondria, and subsequently transfers ubiquitinated‐mitochondria into autophagosomes to initiate mitophagy.^14^ Consequently, triggering mitophagy is an important pathway to block NLRP3 inflammasome activation.

In recent years, the protective effects of flavonoids have aroused a lot of attention in overcoming various diseases, such as cancers, cardiovascular diseases, osteoporosis, and chronic inflammation.^15^ Ononin, a flavonoid isolated from the roots of *Astragalus membranaceus*, has antioxidant, antitumor, and anti‐inflammatory effects.^16^ Although Ononin has been shown to inhibit lipopolysaccharide (LPS)‐induced inflammatory responses, its effect on colitis has not been studied. In the present study, we examined the role of Ononin in mouse colitis induced by dextran sulfate sodium (DSS)‐induced colitis and found that Ononin activated mitophagy, inhibiting the formation of NLRP3 inflammasome and the production of inflammatory cytokines and mediators. Therefore, triggering mitophagy may be a potential strategy for the treatment of inflammatory diseases.

## 2 MATERIALS AND METHODS

### 2.1 Reagents and antibodies

Ononin (99% purit, IO0040) was purchased from Solarbio Science & Technology. Dimethylsulfoxide (DMSO), LPS (*E. coli*: Serotype O55:B5), ATP, poly(dA:dT), and phorbol 12‐myristate 13‐acetate (PMA) were obtained from Sigma‐Aldrich. Muramyl dipeptide and flagellin (Salmonella typhimurium) were obtained from InvivoGen. DSS was obtained from MP Biomedicals Inc. The primary antibodies of IL‐1β, caspase‐1, LC3, SQSTM1/p62, TOM20, β‐actin, and HRP Goat IgG (H+L) were obtained from ABclonal. Diamidino‐phenyl‐indole (DAPI) was brought from Sigma‐Aldrich.

### 2.2 Animals

SPF grade C57BL/6J mice at 6–8 weeks, provided from Beijing Weitong Lihua Experimental Animal Co. Ltd. (SCXK2012‐0001). Mice were fed standard mice chow pellets, water ad libitum, and kept in a laminar flow cabinet with a 12 h light cycles. Experimental protocols were strictly following the ethical regulation of the Committee for Animal Care.

### 2.3 DSS‐induced colitis model

Mice colitis was induced by the administration of DSS in drinking water. The mice received either drinking regular water (control) or 3% (w/v) DSS drinking water (model) for 7 days and thereafter provided with regular water for 3 days. Thirty‐six mice were randomly assigned to normal, DSS‐treated, Ononin (10, 20, or 40 mg/kg)‐treated, and 5‐ASA (40 mg/kg)‐treated groups followed by a randomization procedure (http://www.randomizer.org), with six animals each group. Ononin and 5‐ASA were administered by gavage (0.2 ml) from Day 1 to 10, respectively. The control group was fed with regular water only. The body weight and blood in the stool of the animals were observed once a day. The disease activity index was calculated by assigning well‐established and validated scores as previously described.^17^ On Day 11, following induction with DSS, the animals were killed by CO_2_ inhalation. For histology, segments of the colon taken from the mice were fixed in 10% normal buffered formalin, embedded in paraffin, and stained with hematoxylin and eosin. All experiments are randomized and blinded. The blind will not be uncovered until the data is processed.

### 2.4 Immunofluorescence histochemistry

Paraffin‐embedded colonic tissues were used for the analysis of CD11b macrophage infiltration according to the published procedure.^18^ Briefly, the sections were deparaffinized, rehydrated, and washed in 1% PBS Tween. Then they were treated with 3% hydrogen peroxide, blocked with 3% bovine serum albumin, and incubated for 1 h at room temperature with anti‐CD11b FITC (1:100). The slides were then counter‐stained with DAPI for 30 min. The reaction was stopped by thorough washing in PBS for 5 min. Images were acquired by confocal laser‐scanning microscope (Olympus). Settings for image acquisition were identical for control and experimental tissues.

### 2.5 Cytokine quantification

The distal colon was homogenized in PBS with 0.1 mM benzethonium chloride, 0.05% Tween 20, 10 mM EDTA, and 0.1 mM phenylmethylsulfonyl fluoride as described before.^19^ Tissue homogenates were centrifuged (3000*g*, 15 min) and supernatants containing cytokine profiles were collected. The levels of IL‐1β, IL‐6, IL‐18, and TNF‐α were measured using the ELISA kits according to the relevant protocols.

### 2.6 Cell culture

THP‐1 (Cell Bank of the Chinese Academic of Sciences, Shanghai, China) was cultured in RPMI‐1640 medium (Gibco) containing 10% FBS (Gibco) at 37°C with 5% CO_2_. For THP‐1 cells differentiation, cells were induced by 0.5 mM PMA for 3 h. Then, 500 ng/ml LPS were treated in the absence or presence of Ononin.

### 2.7 Western blot

Western blot analysis was prepared as described previously.^20^ Protein samples were separated by 10%–15% sodium dodecyl‐sulfate polyacrylamide gel electrophoresis and transferred onto nitrocellulose membranes (Millipore). The membranes were blocked with 5% nonfat milk and incubated with indicated primary antibodies overnight at 4°C. After incubated with a horseradish peroxidase (HRP)‐conjugated secondary antibody. Detection was performed using a High‐sig ECL Western Blotting Substrate System.

### 2.8 Measurement of mitochondrial damage

The measurement of mitochondrial damage was performed by flow cytometer using three different stainings. The Mito Tracker Green dye was used to determine the total mitochondrial mass, the Mito Tracker Deep Red was used for mitochondrial membrane potential, and the MitoSOX was applied to measure mitochondrial ROS. All the experiments were conducted by the manufacturer's instructions (YEASEN Biotech). Data were acquired with a FACSCalibur flow cytometer (Becton Dickinson).

### 2.9 Mitochondrial transmembrane potential (ΔΨm) assessment

The differentiated THP‐1 cells were collected and changed to fresh PBS containing JC‐1 for 30 min at 37°C in the dark. After washing three times in PBS, cells were subjected to fluorescence intensity using the FACSCalibur flow cytometer (Becton Dickinson).

### 2.10 Statistical analysis

All experiments are randomized and blinded. Statistical analysis was performed using GraphPad Prism 8. The data were obtained in at least three independent experiments. All results represent mean ± standard deviation and were evaluated by one‐way analysis of variance test. *p* < .05 was considered statistically significant. No data were excluded and outliers were included in the data analysis and presentation.

## 3 RESULTS

### 3.1 Ononin ameliorated DSS‐induced colitis in mice

**Figure 1:**
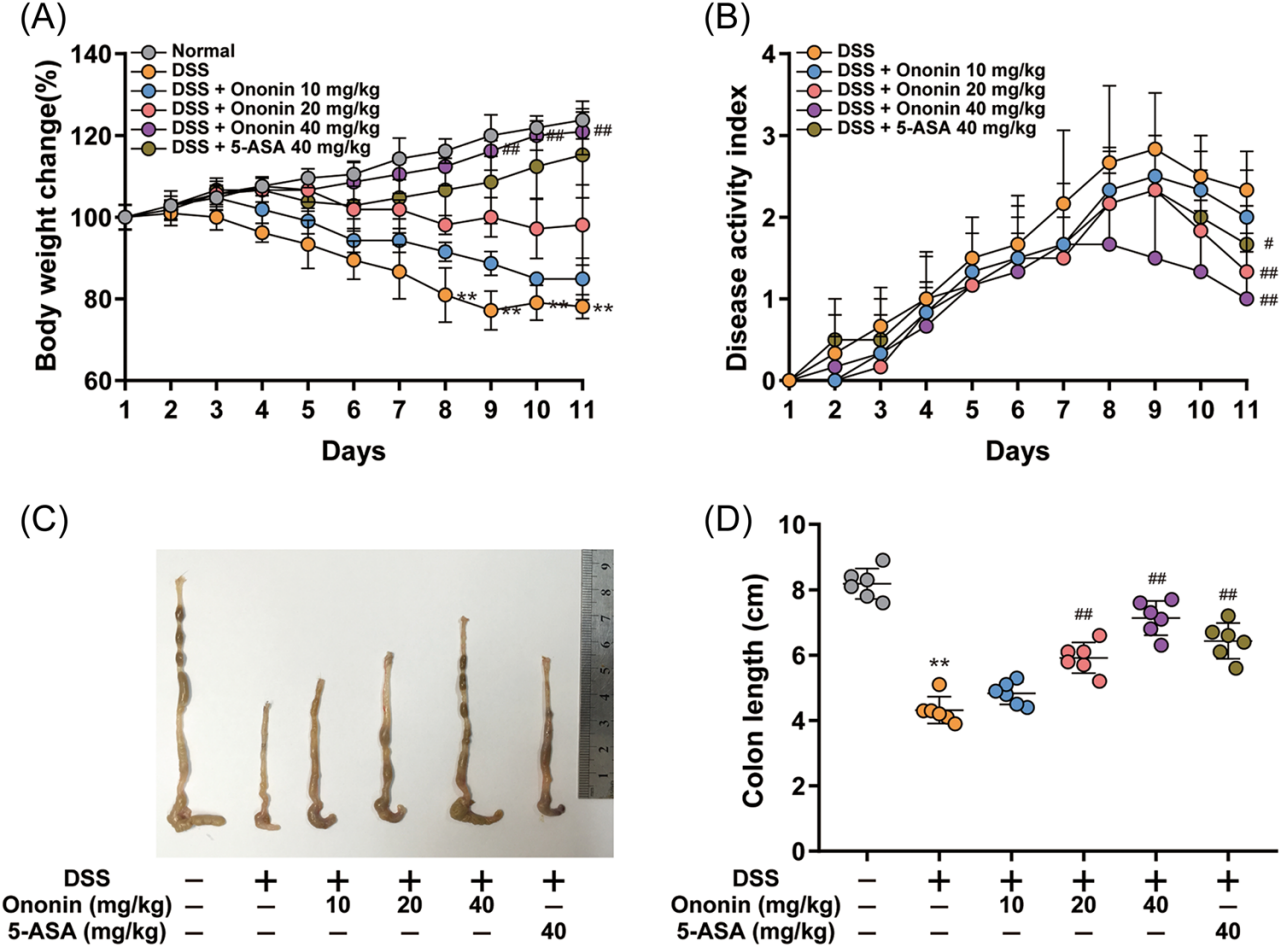
Ononin attenuated DSS‐induced experimental colitis. (A) Body weight changes of each group in DSS‐induced colitis mice. (B) Disease activity index of each group was calculated. (C) The macroscopic appearances and (D) length of colons from each group of mice were measured. Data are presented as mean ± SD. **p < .01 compared with normal group; ^#^ *p* < .05, ^##^ *p* < .01 compared with DSS group. DSS, dextran sulfate sodium.

We established a DSS‐induced colitis model to evaluate the effect of Ononin on IBD. DSS‐induced colitis in mice with obvious diarrhea, severe weight loss, blood in the stool, and shortened colon. Weight loss and disease progression were significantly reversed in the 40 mg/kg Ononin group mice (Figure 1A,B). Colonic morphological results showed that colon length shortening was significantly ameliorated in Ononin (20 or 40 mg/kg) or 5‐ASA (40 mg/kg) group mice (Figure 1C,D). These results suggested that Ononin alleviates colitis induced by DSS in mice.

### 3.2 Ononin ameliorated DSS‐induced inflammatory symptoms in mice

**Figure 2:**
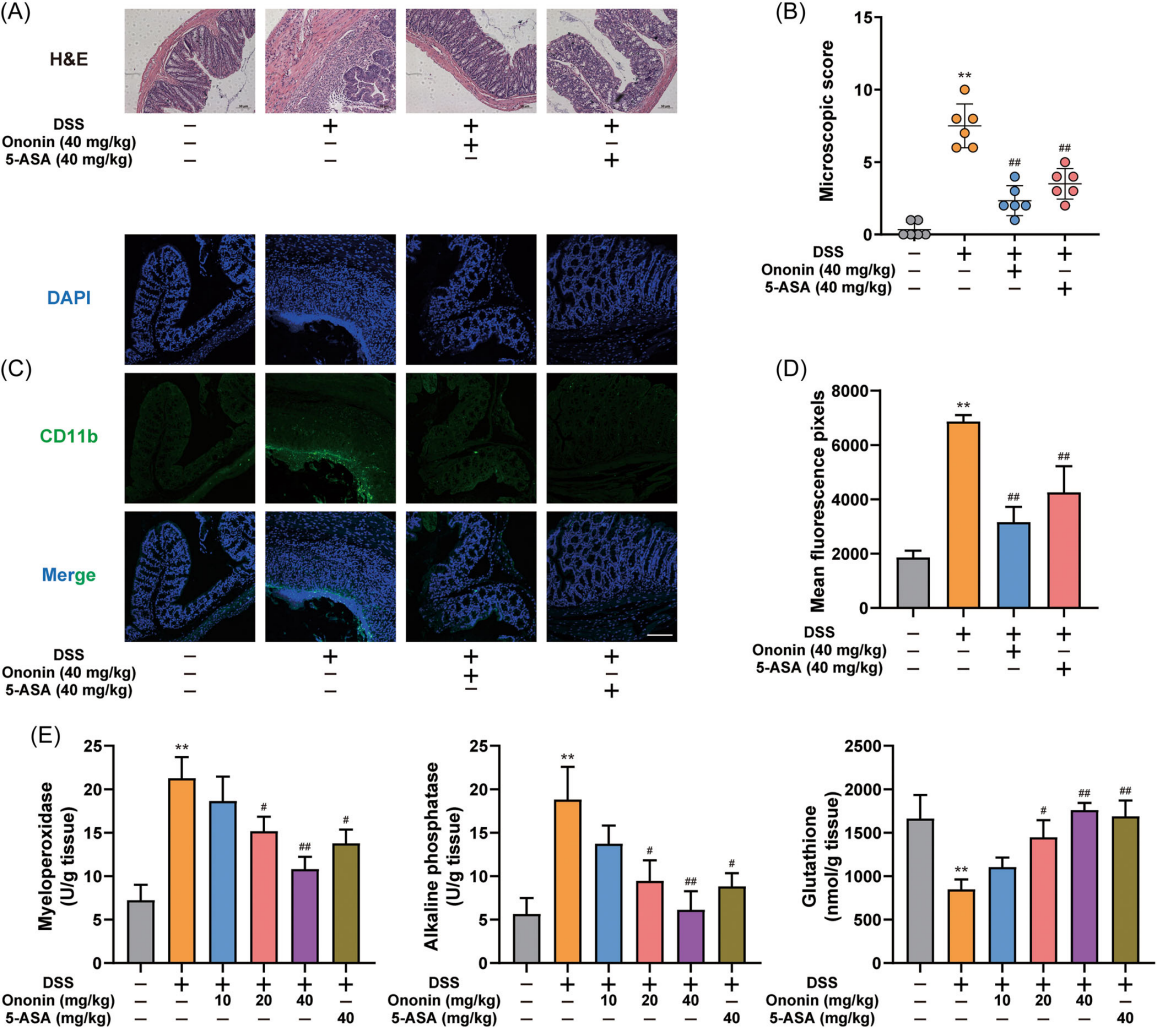
Ononin reduced colon damage in DSS‐induced colitis mice. (A) Serial sections of colon tissues were stained with H&E. (B) Colon histological damage score of each group was determined. (C) Sections of colon tissues were immunostained with DAPI (blue) and anti‐CD11b‐FITC (green) and observed by confocal laser‐scanning microscope. Representative images are shown. Scale bars = 100 µm. (D) Fluorescence intensity of each group was determined. (E) MPO, ALP, and GSH activity in colon samples were determined. Data are presented as mean ± SD. **p < .01 compared with normal group; ^#^ *p* < .05, ^##^ *p* < .01 compared with DSS group. DAPI, diamidino‐phenyl‐indole; DSS, dextran sulfate sodium; H&E, hematoxylin and eosin.

DSS‐induced colitis caused colonic tissue damage and epithelial structure destruction in mice. However, Ononin had a strong protective effect on DSS‐induced colonic inflammation (Figure 2A,B). Given the role of macrophages in intestinal immunity, we examined macrophage infiltration in colon tissue. Compared with the control group, the CD11b positive macrophages were significantly increased in the colon tissue of the DSS group. However, mice treated with Ononin or 5‐ASA showed less macrophage infiltration in colon tissue (Figure 2C,D). Among many proinflammatory mediators, myeloid oxidase (MPO) is positively correlated with the severity of colitis, alkaline phosphatase (ALP) is closely related to the degree of colonic tissue inflammation, and glutathione (GSH) reflects colonic tissue antioxidant level. Our study similarly confirmed that GSH levels were decreased and the ALP and MPO levels were increased in colon tissues of DSS‐treated mice. The ALP and MPO levels were decreased and the GSH levels were increased after treatment with Ononin or 5‐ASA (Figure 2E). These results confirmed that Ononin significantly alleviates DSS‐induced inflammatory symptoms in mice.

### 3.3 Ononin suppressed proinflammatory cytokines production in colon tissue

**Figure 3:**
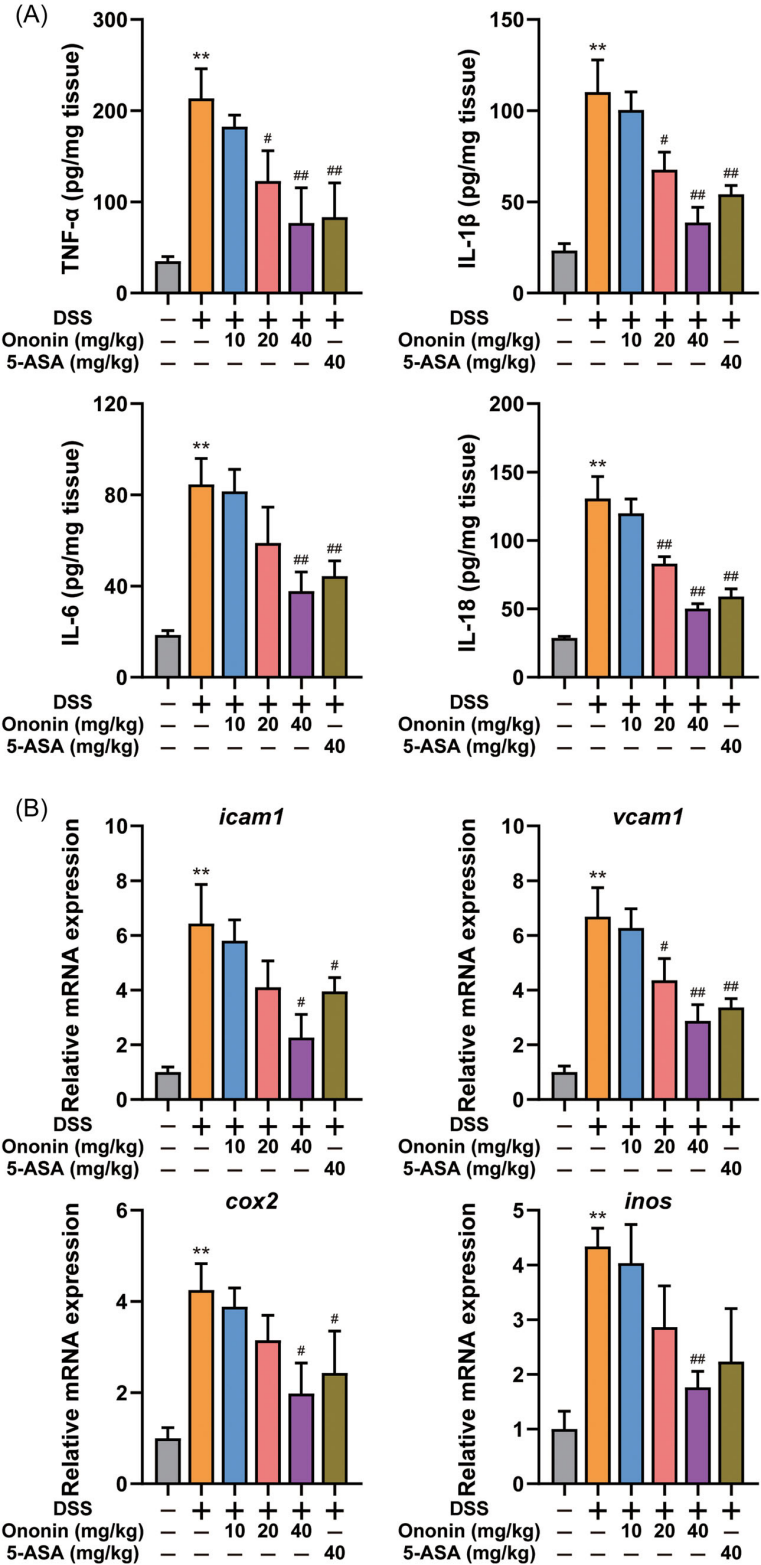
Ononin suppressed proinflammatory cytokine production and reduced the expression of inflammation‐related proteins in colons from mice treated with DSS. (A) The expression levels of cytokines such as TNF‐α, IL‐1β, IL‐6, and IL‐18, in tissue homogenates were detected by ELISA kits. (B) The mRNA expression levels of ICAM1, VCAM1, COX2, and iNOS were determined by quantitative real‐time PCR. Data are presented as mean ± SD. **p < .01 compared with normal group; ^#^ *p* < .05, ^##^ *p* < .01 compared with DSS group. COX2, cycloxygenase‐2; DSS, dextran sulfate sodium; ICAM1, intercellular adhesion molecule‐1; iNOS, inducible nitric oxide synthase; mRNA, messenger RNA; PCR, polymerase chain reaction; VCAM1, vascular cell adhesion molecule‐1.

The production of cytokines associated with colitis was analyzed by ELISA. As shown in Figure 3A, the production of TNF‐α, IL‐1β, IL‐6, and IL‐18 were significantly enhanced in DSS‐treated mice. Ononin or 5‐ASA significantly reduced the production of these cytokines. In addition, the messenger RNA (mRNA) levels of intercellular adhesion molecule‐1 (ICAM1), vascular cell adhesion molecule‐1 (VCAM1), cycloxygenase‐2 (COX2), and inducible nitric oxide synthase (iNOS) were detected. Ononin reduced mRNA levels of these proinflammatory mediators (Figure 3B). These results indicated that the treatment with Ononin could significantly reduce the inflammatory response.

### 3.4 Ononin prevented NLRP3 inflammasome activation

**Figure 4:**
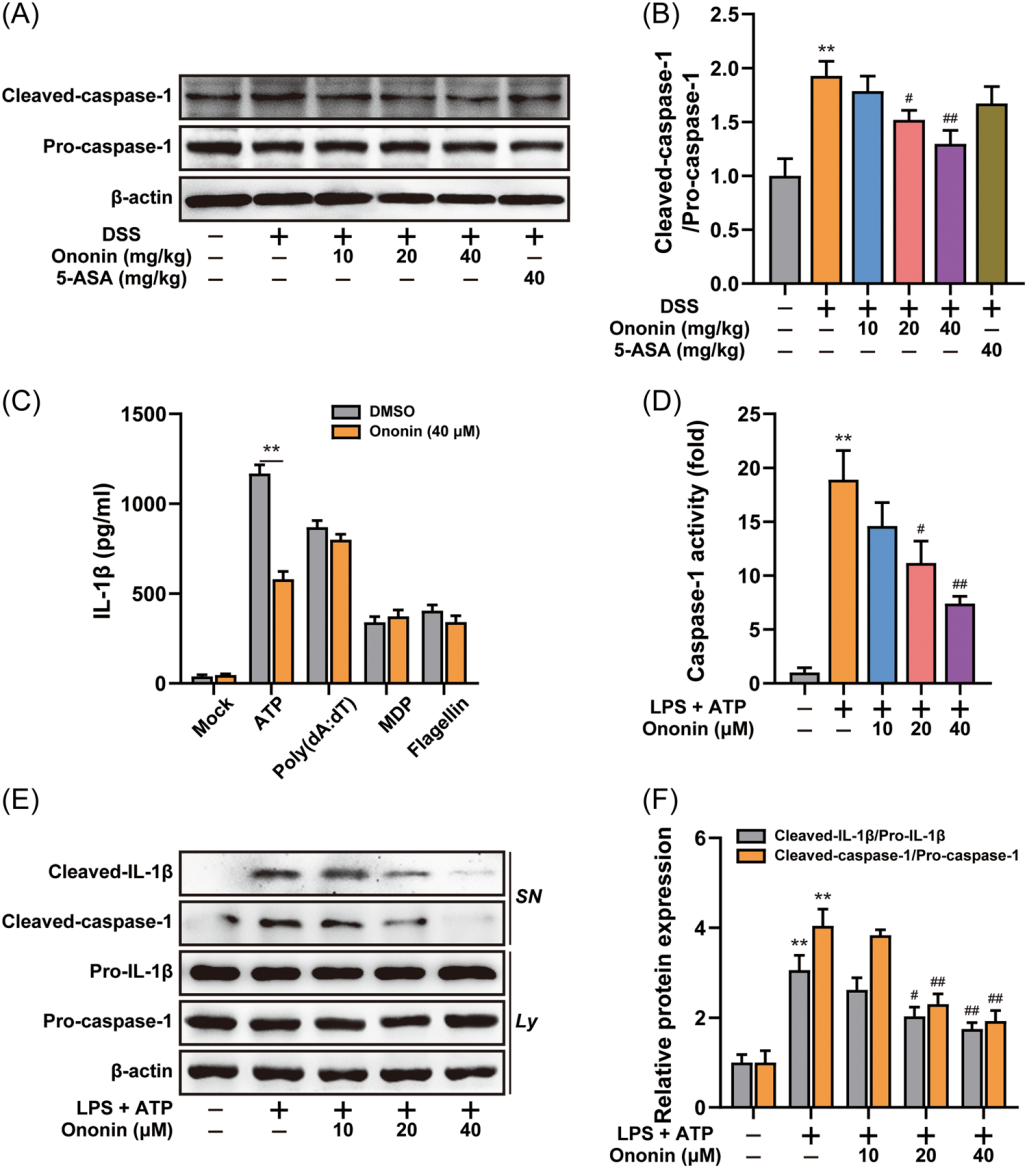
Ononin inhibited activation of NLRP3 inflammasome. (A) Colon tissue protein levels of pro‐caspase‐1 and cleaved‐caspase‐1 were examined by western blot. (B) The quantification of cleaved‐caspase‐1/pro‐caspase‐1 was determined. (C) ELISA of IL‐1β in supernatants of LPS‐primed differentiated THP‐1 cells treated with 40 μM of Ononin, followed by incubation with ATP (5 mM) for 1 h, MDP (200 ng/ml) or flagellin (200 ng/ml) for 6 h, or transfection of poly(dA:dT). (D) The caspase‐1 activity was measured in LPS‐primed differentiated THP‐1 cells treated with Ononin, followed by incubation with ATP (5 mM) for 1 h. (E) Immunoblot analysis of IL‐1β in supernatants (SN) and caspase‐1 in lysate (Ly) of LPS‐primed differentiated THP‐1 cells treated with Ononin, followed by incubation with ATP (5 mM) for 1 h. (F) The quantification of cleaved‐caspase‐1/pro‐caspase‐1 and cleaved‐IL‐1β/pro‐IL‐1β were determined. Data are presented as mean ± SD (*n* = 3). **p < .01 compared with control group; ^#^ *p* < .05, ^##^ *p* < .01 compared with DSS group or LPS plus ATP group. ATP, adenosine triphosphate; DMSO, dimethylsulfoxide; DSS, dextran sulfate sodium; LPS, lipopolysaccharide; MDP, muramyl dipeptide.

It has been studied that the occurrence of colitis is related to the production of IL‐1β by macrophages and IL‐1β maturation is dependent on caspase‐1 activation. Therefore, we investigated the effect of Ononin on caspase‐1 activation. Cleaved caspase‐1 was obviously enhanced by DSS, whereas Ononin reversed this effect (Figure 4A,B). The assembly of inflammasomes activates caspase‐1, so we investigated the effect of Ononin on the activation of different inflammasomes. We found that Ononin inhibited IL‐1β secretion induced by NLRP3 inflammasome agonists, but had no effect on other inflammasome agonists (Figure 4C). Consistently, Ononin reduced caspase‐1 activity triggered by NLRP3 inflammasome agonists (Figure 4D). In addition, Ononin obviously reduced IL‐1β and cleaved caspase‐1 expression in the supernatant (Figure 4E,F). This suggested that Ononin blocked NLRP3 inflammasome activation.

### 3.5 Ononin alleviated mitochondrial damage induced by NLRP3 agonist

**Figure 5:**
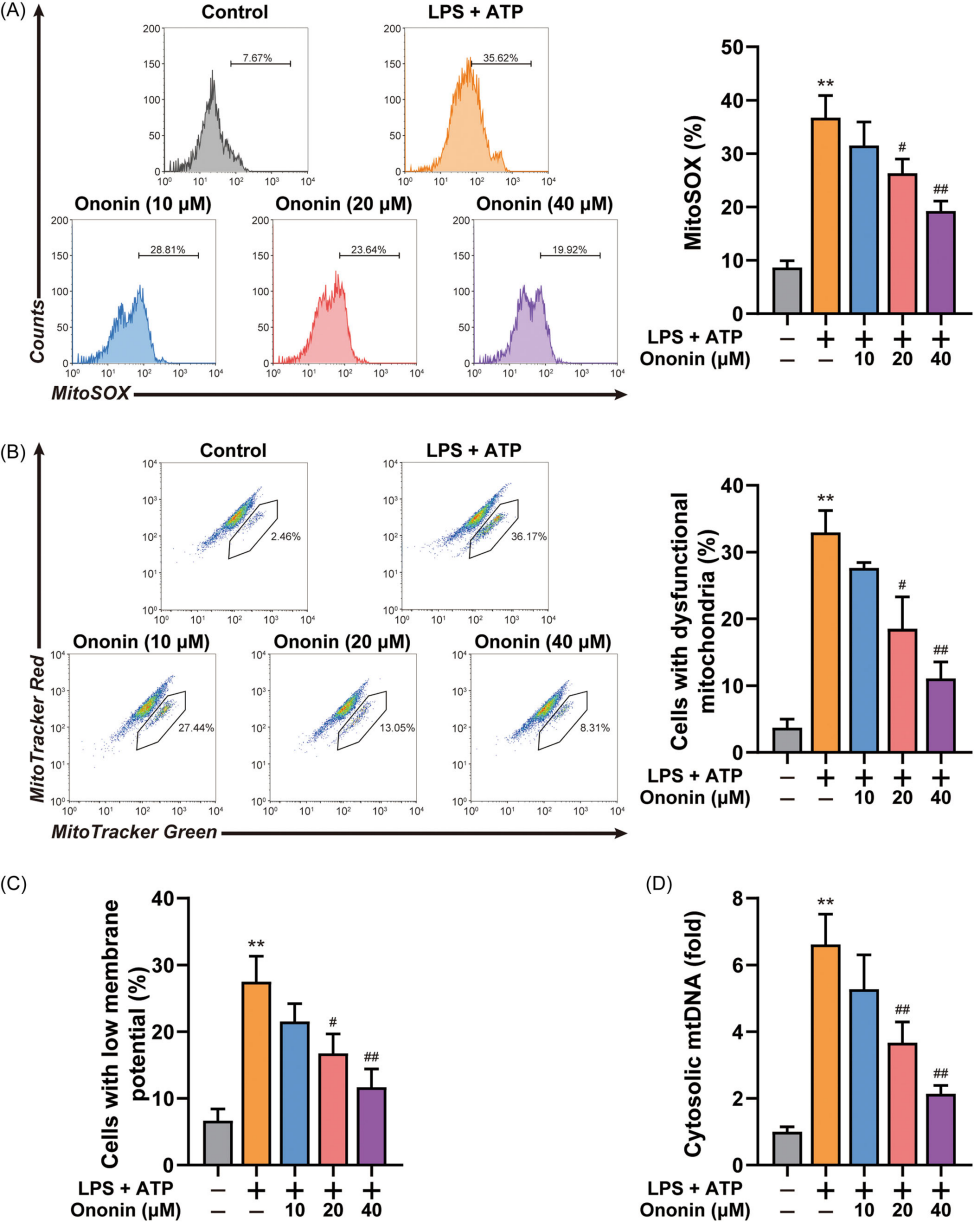
Ononin reduced accumulation of damaged mitochondria. (A) Flow cytometry analysis (left) and quantification (right) of mitochondrial ROS by MitoSOX staining in LPS‐primed differentiated THP‐1 cells treated with Ononin, followed by stimulation with ATP (5 mM) for 1 h. (B) Flow cytometry analysis (left) and quantification (right) of mitochondrial status in LPS‐primed differentiated THP‐1 cells treated as above. Gates represent cells with damaged mitochondria. *p < .05, **p < .01. (C) Flow cytometry analysis of mitochondrial membrane potential by JC‐1 staining in LPS‐primed differentiated THP‐1 cells treated as above. **p < .01. (D) Quantitative real‐time PCR analysis of mtDNA released from LPS‐primed differentiated THP‐1 cells treated as above. Data are presented as mean ± SD (*n* = 3). **p < .01 compared with control group; ^#^ *p* < .05, ^##^ *p* < .01 compared with LPS plus ATP group. ATP, adenosine triphosphate; LPS, lipopolysaccharide; mtDNA, mitochondrial DNA; PCR, polymerase chain reaction.

NLRP3 inflammasome activation is a result of mitochondrial damage. Our study similarly found that mtROS production, mitochondrial dysfunction, and membrane potential loss in response to ATP, while Ononin reversed mitochondrial damage (Figure 5A–C). Moreover, Ononin also reduced mtDNA release (Figure 5D). Thus, Ononin attenuated mitochondria damage induced by NLRP3 agonists.

**Figure 6:**
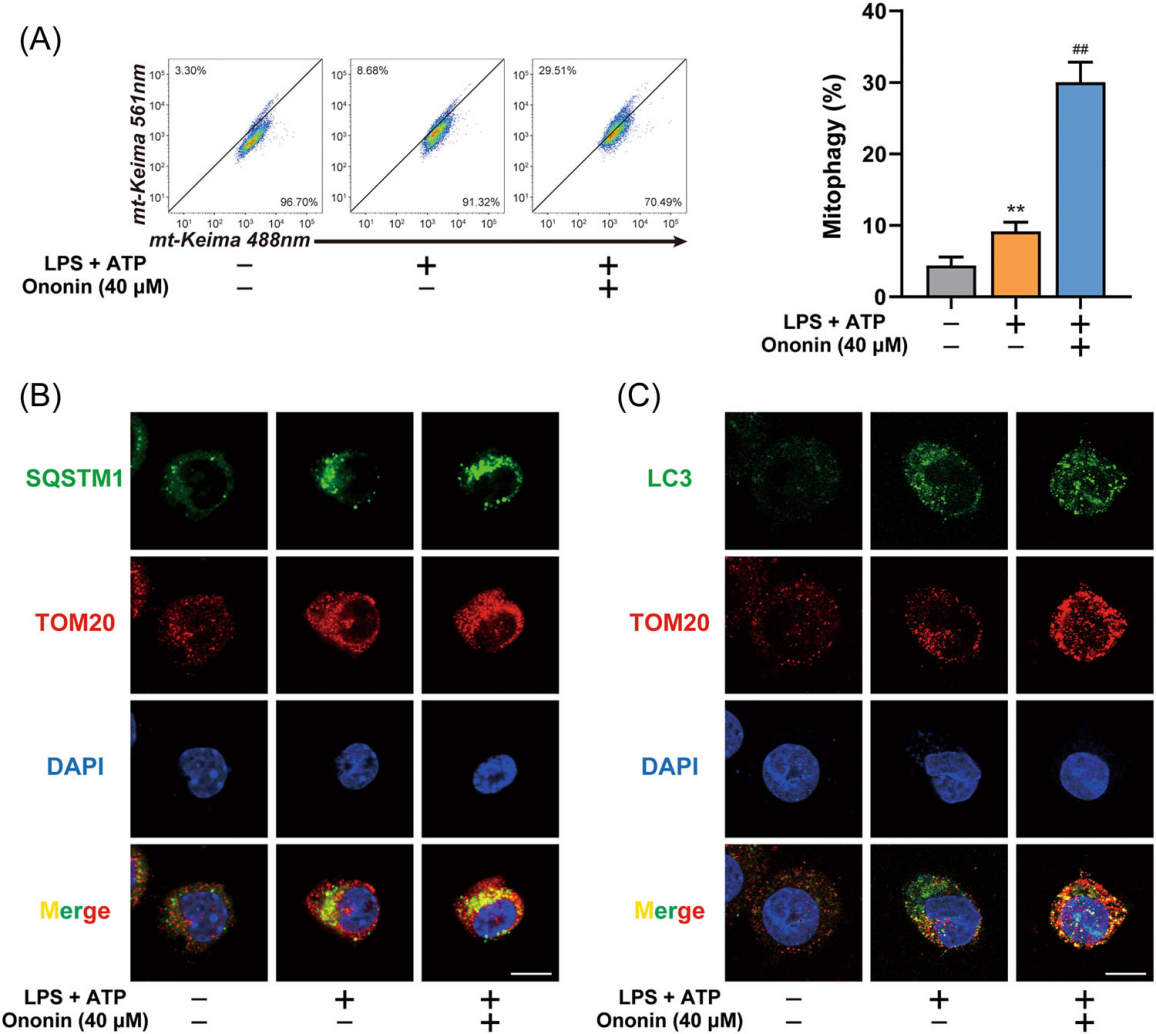
Ononin triggered mitophagy to clear damaged mitochondria. (A) FACS analysis (left) and quantification (right) of LPS‐primed THP‐1 cells expressing mito‐Keima treated with 40 μM of Ononin, followed by stimulation with ATP (5 mM) for 1 h. (B, C) Intracellular distribution of SQSTM1 (B) or LC3 (C) and mitochondria (TOM20) in LPS‐primed THP‐1 cells treated with 40 μM of Ononin, followed by stimulation with ATP (5 mM) for 1 h, examined by confocal microscopy. Scale bars = 10 µm. Data are presented as mean ± SD (*n* = 3). **p < .01 compared with control group; ^##^ *p* < .01 compared with LPS plus ATP group. ATP, adenosine triphosphate; LPS, lipopolysaccharide.

It was demonstrated that mitophagy inhibits NLRP3 inflammasome by clearing damaged mitochondria. We examined the effect of Ononin on mitophagy and found that Ononin promoted the translocation of mitochondria to lysosomes (Figure 6A). In addition, we observed that Ononin promoted the colocalization of SQSTM1 and LC3 with mitochondria (Figure 6B,C). This suggested that Ononin promoted mitophagy during the activation of the NLRP3 inflammasome.

### 3.6 Mitophagy was involved in Ononin‐suppressed NLRP3 inflammasome

**Figure 7:**
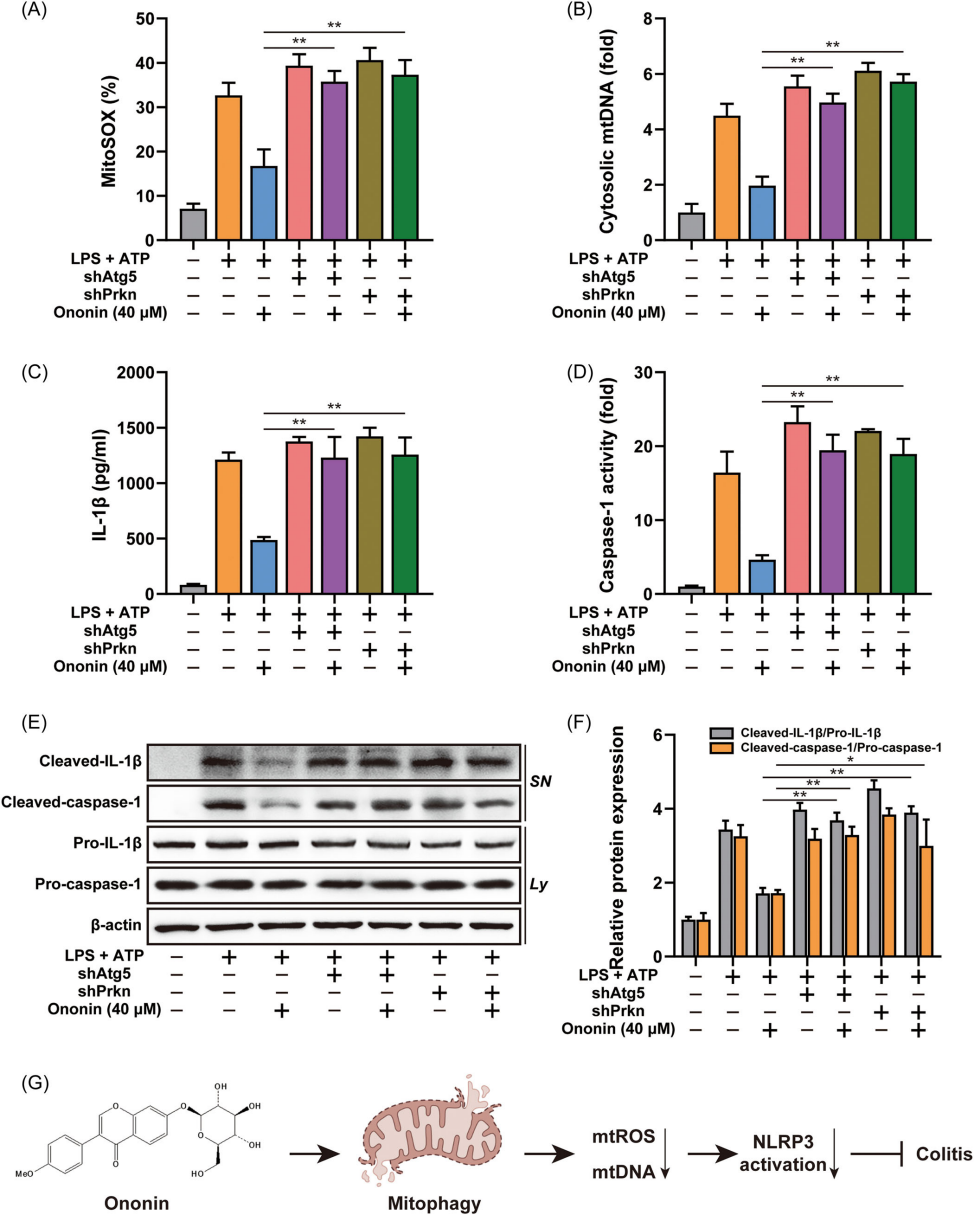
Mitophagy is involved in the inhibition of NLRP3 inflammasome mediated by Ononin. (A) Quantification of MitoSOX staining in LPS‐primed THP‐1 cells stably expressing *Atg5 or Prkn* shRNA, which was treated with 40 μM of Ononin, followed by stimulation with ATP (5 mM) for 1 h. (B) Quantitative real‐time PCR analysis of mtDNA released from LPS‐primed differentiated THP‐1 cells stably expressing *Atg5 or Prkn* shRNA treated as above. (C) ELISA of IL‐1β in supernatants of LPS‐primed differentiated THP‐1 cells stably expressing *Atg5 or Prkn* shRNA treated as above. (D) The caspase‐1 activity was measured in LPS‐primed differentiated THP‐1 cells stably expressing *Atg5 or Prkn* shRNA treated as above. (E) Immunoblot analysis of IL‐1β in supernatants (SN) and caspase‐1 in lysate (Ly) of LPS‐primed differentiated THP‐1 cells stably expressing *Atg5 or Prkn* shRNA treated as above. (F) The quantification of cleaved‐caspase‐1/pro‐caspase‐1 and cleaved‐IL‐1β/pro‐IL‐1β were determined. (G) The flowchart to show the whole experimental design. Data are presented as mean ± SD (*n* = 3). *p < .05, **p < .01. ATP, adenosine triphosphate; LPS, lipopolysaccharide; mtDNA, mitochondrial DNA; mtROS, mitochondrial reactive oxygen species; PCR, polymerase chain reaction; shRNA, short hairpin RNA.

To elucidate the relationship between Ononin blocking NLRP3 inflammasome activation and promoting mitophagy, we silenced ATG5 or Parkin expression. We found that either the reduced release of mtROS and mtDNA (Figure 7A,B) or the suppressed IL‐1β secretion and caspase‐1 activity by Ononin (Figure 7C,D) was significantly reversed by knockdown of ATG5 or Parkin. Even the downregulation of IL‐1β and cleaved caspase‐1 in the supernatant by Ononin was also nearly abolished by knockdown of ATG5 or Parkin (Figure 7E,F). This suggested that Ononin suppressed NLRP3 inflammasome by mitophagy.

## 4 DISCUSSION

IBD is a chronic recurrent inflammatory disease, and long‐term chronic inflammation can eventually progress to colorectal cancer.^21^ There is no effective treatment for IBD. At present, conventional treatment is the main clinical treatment. Treatment options include immunosuppressive agents, anti‐inflammatory drugs, and biologic therapies that target inflammatory pathways. Although these treatments alleviate the development of IBD to some extent, up to 40% of patients have no effect.^22^ Therefore, there is an urgent need to develop novel, safe, and effective drugs.

Ononin is a flavonoid isolated from *A. membranaceus* root. Astragalus polysaccharide and Astragalus saponins were reported to have a protective effect on colitis in *A. membranaceus*, but the effect of Ononin on colitis has been poorly studied.^15^ Our results find that Ononin may alleviate colitis by inhibiting macrophage NLRP3 inflammasome. The symptoms of mice in DSS‐treated colitis contain diarrhea and bloody stools. However, mice treated with Ononin show different results, such as reduced body weight loss, shorten colon length, and decrease colon injuries. Macrophages are directly involved in the pathogenesis of IBD. Our study confirms that Ononin inhibits CD11b‐positive macrophage infiltration in DSS‐induced colitis tissues. In addition, we subsequently investigate the effect of Ononin on macrophage using human THP‐1 cells in vitro. Furthermore, our results support that Ononin reduces ALP and MPO production and enhanced GSH levels in DSS‐induced colitis mice. During the colitis, inflammatory cytokines are secreted and involved in inflammatory cascades. It also produces a variety of inflammatory mediators such as VCAM1, ICAM1, iNOS, and COX2 in inflamed colon tissues. Ononin could inhibit the production of inflammatory cytokines and reduce the mRNA levels of inflammatory mediators in the colon tissues of mice after DSS treatment (Figure 3).

NLRP3 inflammasome activation in macrophages plays an important role in the progression of inflammation. The NLRP3 inflammasome, which consists of NLRP3, ASC, and caspase‐1, is critical for the maturation of IL‐1β and IL‐18.^23^ Assembly of the NLRP3 inflammasome promotes cleavage of caspase‐1 to gain the ability to cleave IL‐1β and IL‐18.^24^ NLRP3 inflammasome activation plays an important role in colitis. Colitis has been shown to be less severe in NLRP3‐deficient mice than in wild‐type mice. In addition, inhibitors of the NLRP3 inflammasome significantly alleviated colitis symptoms. Our results support that Ononin inhibits the maturation and secretion of IL‐1β induced by NLRP3 agonists (Figure 4). Moreover, in colonic tissues of mice with colitis, Ononin also inhibits caspase‐1 activation in a dose‐dependent manner.

Mitochondrial damage could lead to NLRP3 inflammasome activation.^25^ In normally functioning macrophages, damaged mitochondria are usually degraded by mitophagy.^26^ However, this balance will be disrupted under continuous conduction of NLRP3 signaling. In the end, mitochondrial damage occurs, which causes the release of mtDNA and mtROS, and ultimately activated the NLRP3 inflammasome. In this process, mitochondrial damage is critical for regulating NLRP3 inflammasome activation. While, Ononin inhibits the occurrence of mitochondrial damage (Figure 5).

Given the role of mitophagy in NLRP3 inflammasome activation, our results support that Ononin significantly enhances mitophagy levels (Figure 6).^27^ The removal of damaged mitochondria by mitophagy requires two steps. First, mitophagy initiation depends on the recruitment of PINK1 and Parkin to damaged mitochondria.^28^ Parkin promotes the ubiquitination of mitochondrial outer membrane, and then triggers the binding of autophagy receptors to ubiquitinated mitochondria membrane to initiate mitophagy.^29^ Then, specific autophagy mechanisms are activated to degrade mitochondria. ATG5 regulates LC3 lipidation through formation of ATG12–ATG5–ATG16 complex, which plays a key role in regulating autophagy activity.^30^ Therefore, our study confirmed that Ononin inhibits NLRP3 inflammasome by triggering mitophagy by knockdown of Atg5 or Parkin, respectively. The discovery of mitophagy activation provides a new idea for the study of Ononin's anti‐colitis mechanism, and provides the possibility for further research on drugs for IBD therapy (Figure 7G).

## AUTHOR CONTRIBUTIONS

**Ting Yu**: conceptualization; investigation; methodology; project administration; resources; supervision; visualization; writing – review & editing. **Xuejia Lu**: investigation; validation. **Yan Liang**: investigation; methodology; project administration. **Lin Yang**: investigation; project administration. **Yuehan Yin**: data curation; visualization. **Hong Chen**: supervision; validation; writing – original draft.

## CONFLICT OF INTEREST STATEMENT

The authors declare no conflict of interest.
